# Juvenile-onset *PSAT1*-related neuropathy: A milder phenotype of serine deficiency disorder

**DOI:** 10.3389/fgene.2022.949038

**Published:** 2022-08-16

**Authors:** Yu Shen, Yun Peng, Pengcheng Huang, Yilei Zheng, Shumeng Li, Kaiyan Jiang, Meihong Zhou, Jianwen Deng, Min Zhu, Daojun Hong

**Affiliations:** ^1^ Department of Neurology, The First Affiliated Hospital of Nanchang University, Nanchang, China; ^2^ Department of Medical Genetics, The First Affiliated Hospital of Nanchang University, Nanchang, China; ^3^ Department of Neurology, Peking University First Hospital, Beijing, China

**Keywords:** serine deficiency disorder, *PSAT1* gene, ichthyosis, peripheral neuropathy, juvenile-onset

## Abstract

**Background:** Primary serine deficiency disorders have a broad range of the phenotypic spectrum. As an inborn error of metabolism, individuals with severe phenotype may be easily recognized with Neu-Laxova syndrome. However, late-onset mild phenotypes may be underdiagnosed and will lead to disastrous consequences due to treatment delays.

**Materials and Methods:** Clinical features of patients with serine deficiency disorders were summarized in two unrelated patients. Skin and sural nerve biopsies were conducted on the patients. Whole exome sequencing (WES) was performed in the index patients. Sanger sequencing was used to analyze family cosegregation.

**Results:** Patient 1 was a 19-year-old male presenting with infancy-onset ichthyosis and juvenile-onset neuropathy. Patient 2 was a 17-year-old male manifesting childhood-onset ichthyosis and juvenile-onset neuropathy. Except for nystagmus, no other developmental or neurodegenerative disorders were found in the patients. Electrophysiological studies indicated a severe sensorimotor axonal neuropathy with a possible demyelinating component. High-dose oral L-serine and glycine completely alleviated skin lesions and only slightly improved neuropathy symptoms. Skin biopsies showed typical features consistent with ichthyosis and severe loss of unmyelinated axons. Sural biopsies revealed a severe loss of axons and a few thinly myelinated fibers. WES found the same homozygous variant c.43G > C (*p*.A15P) in the *PSAT1* gene, which was cosegregated in the two families.

**Conclusions:** The skin and nervous system may be the main affected targets in serine deficiency disorders. Our patients show a more simple and mild phenotype of *PSAT1*-related serine deficiency disorder. The pathological changes and regenerative ability of skin and peripheral nerves determine their response to serine supplements.

## 1 Introduction

**FIGURE 1:**
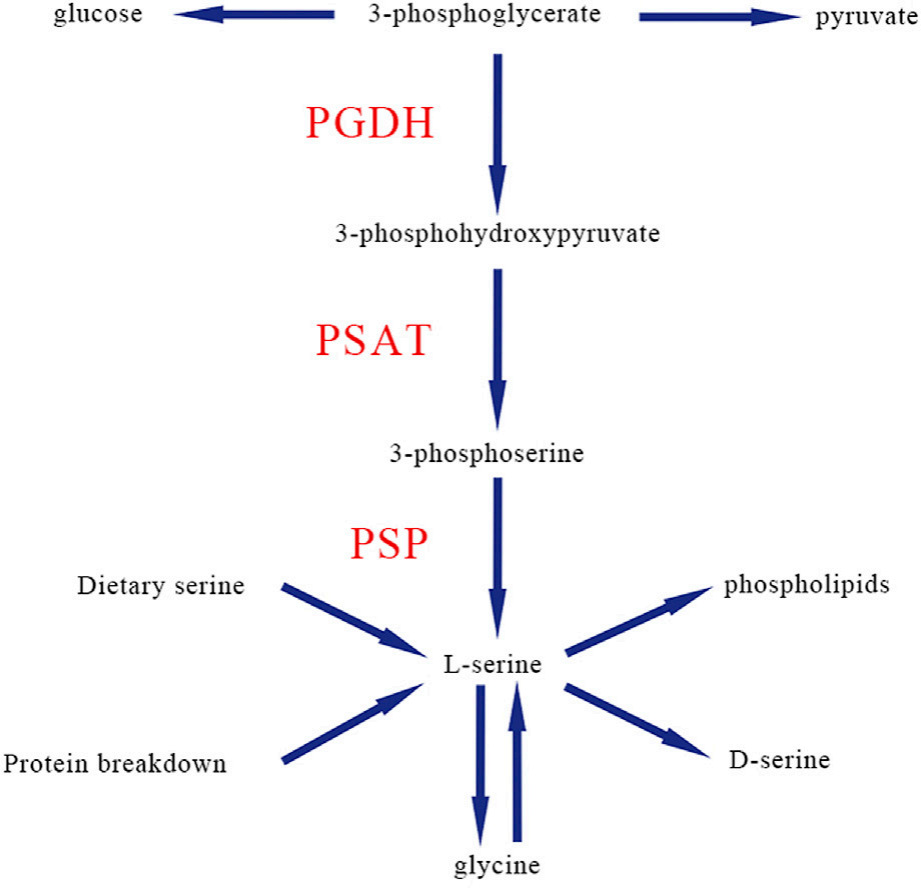
Classic biosynthesis pathway of *de novo* L-serine in human. PGDH, phosphoglycerate dehydrogenase; PSAT, phosphoserine aminotransferase; PSP, and phosphoserine phosphatase.

Serine deficiency disorders include a group of inherited metabolic diseases caused by a defect in one of the three synthetases in the L-serine biosynthesis pathway (Jaeken et al., 1996). L-serine, as a nonessential amino acid (van der Crabben et al., 2013), is biosynthesized through a triad of enzymes including phosphoglycerate dehydrogenase (PGDH; encoded by the *PHGDH* gene), phosphoserine aminotransferase (PSAT; encoded by the *PSAT1* gene), and phosphoserine phosphatase (PSP; encoded by the *PSPH* gene) (Cheung et al., 1969; Rowsell et al., 1969; Snell, 1985; Tabatabaie et al., 2010). The first step of serine biosynthesis is that 3-phosphoglycerate is converted into 3-phosphohydroxypyruvate by PGDH, then the second is converted to 3-phosphoserine by PSAT, and finally is removed from the phosphate group to form L-serine by PSP (Figure 1).

Primary defects in all three enzymes of serine biosynthesis have been associated with a broad range of the phenotypic spectrum (El-Hattab et al., 2016). Currently, the most severe end of the spectrum is Neu-Laxova syndrome that is characterized by prenatal growth deficiency with microcephaly, brain malformation, arthrogryposis, ichthyosis, and characteristic facial features including ectropion and eclabion (Acuna-Hidalgo et al., 2014; Mattos et al., 2015; Vincent et al., 2015). Milder individuals with infantile serine biosynthesis defects exhibit intrauterine growth restriction with microcephaly, spastic tetraplegia, seizures, psychomotor arrest, brain atrophy with hypomyelination, and anemia (Hart et al., 2007; Tabatabaie, et al., 2010; El-Hattab, et al., 2016). The mild end of this spectrum is childhood serine biosynthesis defect, which presents with developmental delay and intellectual disability of variable severity, epilepsy, ataxia, hypertonia, ichthyosis, and progressive peripheral neuropathy (Debs et al., 2021).

PSAT deficiency was first reported in two siblings, and the index patient showed intractable seizures, microcephaly, hypertonia, and psychomotor retardation and died at the age of 7 months (Hart, et al., 2007). *PSAT1* was subsequently investigated as a candidate gene for serine metabolic dysfunction, and mutations in *PSAT1* were found in six individuals with Neu-Laxova syndrome (Acuna-Hidalgo, et al., 2014).In this study, we reported two independent patients with the same homozygous mutation who presented with ichthyosis of variable severity and juvenile-onset peripheral neuropathy. Our study expanded the clinical spectrum of *PSAT1*-related serine deficiency.

## 2 Materials and Methods

### 2.1 Subjects

Patients with *PSAT1* mutations were recruited from a tertiary general hospital. A detailed medical history was obtained from the subjects and their relatives. Information regarding the age of onset, progression of the disease, family history, and other clinical manifestations was collected. A battery of clinical and laboratory investigations were conducted to investigate the inflammatory, toxic, or metabolic origins. An electrophysiological study was performed on the nerves using a standard method with surface electrodes for stimulation and recording. Blood and urine samples before treatment from the two patients were collected for amino acids, acylcarnitine, and organic acid analysis.

All tissue samples were obtained after a written consent signed by each individual in compliance with the bioethics laws of China as well as the Declaration of Helsinki. The research was approved by ethics committee of the first affiliated hospital of Nanchang University.

### 2.2 Skin and sural nerve biopsies

Skin and sural nerve biopsies in the distal part of the leg were conducted on the two patients. For electron microscopy, a part of the specimens was initially fixed in 2.5% glutaraldehyde, subsequently fixed in 1% osmium tetroxide, and embedded in Epon 812. Ultrathin sections were contrasted with uranyl acetate and lead citrate before being examined under electron microscopy (JEOL-1230, Japan). The rest of the specimens were fixed by 4% formalin solution, embedded in paraffin, cut into 4-mm thick sections, and stained with hematoxylin and eosin (H & E). Immunostaining with neurofilament (NF) and myelin basic protein (MBP) antibodies was performed to observe the axon and myelin sheath, respectively.

### 2.3 Genetic screening

Genomic DNA was extracted from peripheral blood samples. Whole exome sequencing (WES) was commercially supported by Running Gene Inc. (Beijing, China). In brief, targeted exon enrichment was performed using Sure Select Human All Exon V5 (Agilent Technologies). The exon-enriched DNA libraries were subjected to paired-end sequencing with the Hiseq2000 platform (Illumina, Inc.). The sequenced data were aligned to the human reference genome (hg19) using the Burrows-Wheeler Aligner (BWA) (Li and Durbin, 2009). Duplicate reads were removed by Picard tools. The Genome Analysis Toolkit (GATK) was used to call the single-nucleotide polymorphism (SNPs) and insertions/deletions (indels) following the best practices. The variants were subsequently annotated with Annovar. Variations obtained from exome sequencing with minor allele frequencies >0.05 in public databases (1000 Genomes project, dbSNP, ExAC, and gnomAD) were excluded. Multiple prediction software programs were used to analyze the effect of single-nucleotide variants (SNVs) as previously described: SIFT, PolyPhen-2, Mutation Taster, and REVEL. We then performed variant classification according to American college medical genetics and genomics criteria (ACMG) guidelines. Sanger sequencing with specific primers was conducted to confirm the *PSAT1* mutation in the patients and their available family members.

## 3 Results

### 3.1 Clinical features

#### 3.1.1 Patient one

The individual was a 19-year-old male from a nonconsanguineous family and had a healthy sister 8 years older than him. The patient was born with birth weight, length, and head circumference within the standard percentile. He also had normal developmental milestones. Flaky and scaling skin on the trunk and limbs had been noticed since infancy, which obviously deteriorated in winter and slightly relieved in summer or hot weather. At age 16, the patient found himself walking on his toes and his heels could not touch the ground. He complained of frequent stumbles on uneven ground and numbness in distal lower limbs. One year later, he noticed high arches on his feet. The sensory disturbance gradually developed upward. He also had difficulty walking quickly and keeping on steady. At age 19, the patient had muscle weakness in both thumbs that showed difficulty in straightening, writing, and picking up food. He also showed difficulty in climbing the stairs and standing up after squatting.

**FIGURE 2:**
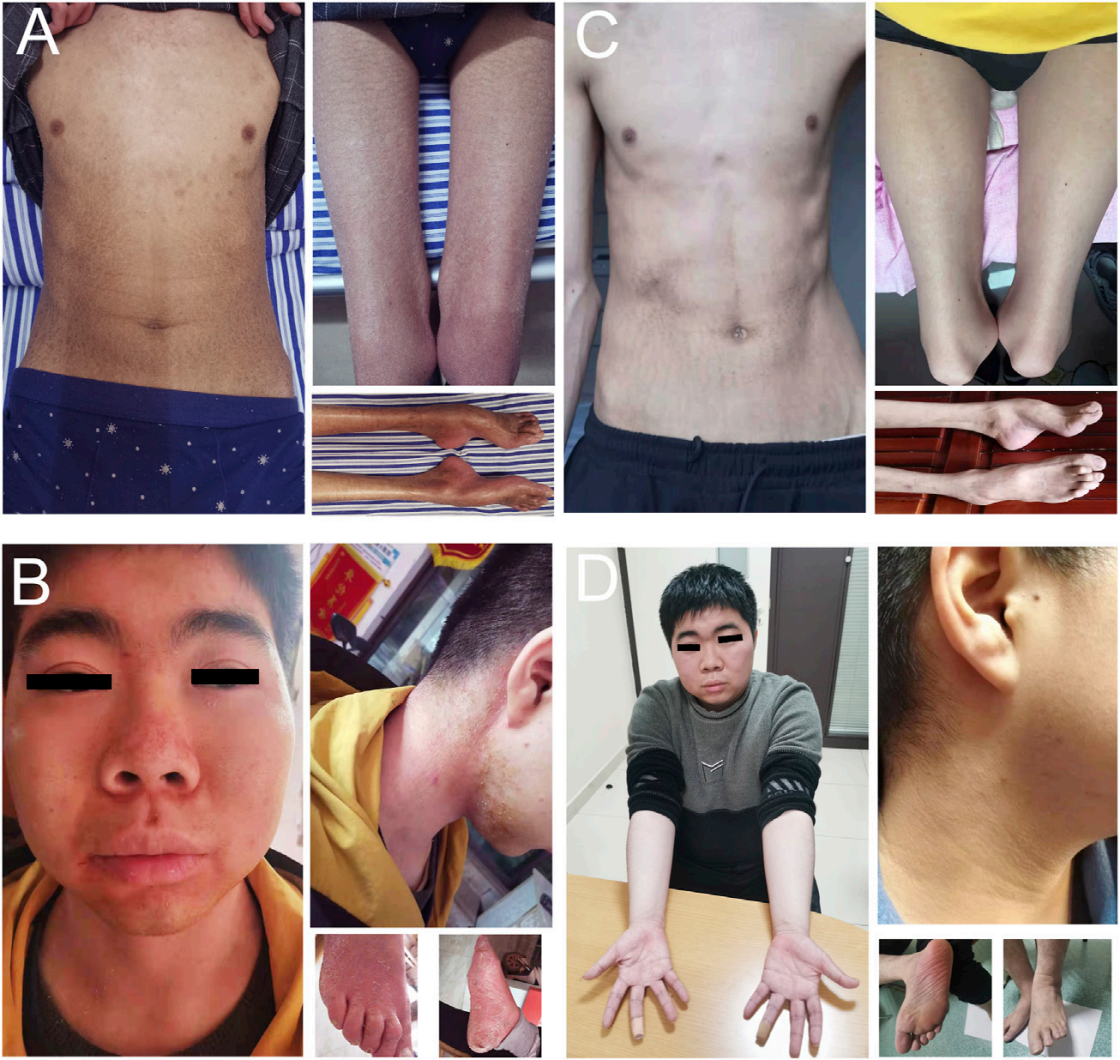
Skin changes in PSAT1-related patients before and after L-serine treatment. Patient 1 showed generalized xerotic skin with fine scaling and areas with a shiny and atrophic texture in his upper forearms, upper chest, abdomen, thighs and foot **(A)**. Patient 2 showed red skin and mildly swelling on the face, hands, and feet, as well as ichthyosiform scaling in his neck and feet **(B)**. After L-serine and glycine treatment for 3–5 months, the skin was completely recovered in patient 1 **(C)** and patient 2 **(D)**.

The patient at age 19 had a normal cognitive function with 30 scores on the mini-mental state examination (MMSE). The body mass index (BMI) was 14.2 (normal 18.5–23.9). Physical examination revealed generalized xerotic skin with fine scaling and areas with a shiny and atrophic texture. Ichthyosiform scaling was prominent in his upper forearms, upper chest, abdomen, and thighs (Figure 2A). The hands and feet showed thin and reddish skins. Pes cavus and contracture of the Achilles tendon were observed. Obvious atrophy of the lower limbs and mild wasting of interosseous muscles of the hands were also observed. Muscle strength of foot dorsiflexors graded 2/5 on the Medical Research Council (MRC) score. Peroneus muscles and the muscles of plantar flexion were graded 3/5. Muscle strength of proximal lower limbs graded 4/5. In the upper limbs, interosseous and extensor muscles of hands graded 2/5, and thenar and hypothenar muscles graded 4/5. The proximal muscles of the upper limbs were normal. Pain, light touch, vibration, and joint position perception were reduced below the knee or the wrists. Deep tendon reflexes were not elicited in the lower limbs and were reduced in the upper limbs. Mild horizontal and vertical nystagmus was noticed.

Blood count, blood biochemistry, serum creatine kinase (CK), inflammatory tests, paraneoplastic antibody panel, thyroid hormones, serum vitamin B12, and folic acid were within normal limits. Immunofixation electrophoresis was negative. The panel of anti-ganglioside antibodies including GQ1b, GT1b, GD1b, GD1a, GM2, and GM1 was negative. Cerebrospinal fluid (CSF) results were normal. Blood acylcarnitine and urine organic acid analysis were normal. Plasma L-serine was 66.6 μmol/L (normal 65–180 μmol/L) at 12 h after fasting and other amino acids were within normal limits. Brain magnetic resonance imaging (MRI) and MR angiography (MRA) were normal. An electroencephalogram (EEG) showed no abnormalities. A pure hearing threshold examination revealed a decrease in perception of low-frequency sounds (125–500 Hz). Video-head impulse test indicated that the patient had impairments of bilateral horizontal and right posterior semicircular canals. Complete ocular examinations were normal.

#### 3.1.2 Patient two

The patient is a 17-year-old male from a consanguineous family. He was born with normal head circumference, weight, and length. At age 4, the patient showed reddish and flaky skin on the plantar and toes, which gradually developed upward thighs and hands. The skin lesions were aggravated in winter and spring and mildly alleviated in summer and autumn. At age 16, the skin of the face, neck, and distal limbs is significantly reddened, desquamated, and locally ulcerated. Meanwhile, the patient gradually felt weakness in both the lower limbs and could not walk on tiptoe, but could walk with heels. Recently, he showed difficulty in climbing the stairs and standing up after squatting. He also felt numbness in his feet. Nail malnutrition, fingertip, and plantar ulcers were found.

The patient at age 17 had a normal cognitive function with 30 scores of MMSE. The BMI was 22.5. The skin was red and mildly swollen on the face, hands, and feet. Ichthyosiform scaling was prominent in his neck, abdomen, and feet (Figure 2B). Muscle strength grade was 5/5 in the upper limbs, 4/5 in the proximal lower limbs, 3/5 in the foot dorsiflexion, and 4/5 in the foot plantar flexion. Pain and temperature sensations were reduced below the ankles. Deep sensation was normal. Deep tendon reflexes were normal in the upper limbs, reduced at the knee, and diminished at the ankles. Mild horizontal and vertical nystagmus was noticed.

Blood count, blood biochemistry, serum CK, inflammatory tests, paraneoplastic antibody panel, thyroid hormones, serum vitamin B12 and folic acid were within normal limits. Immunofixation electrophoresis was negative. The panel of antiganglioside antibodies was negative. The protein level of CSF was 534.4 mg/L (normal 450–150 mg/L). Blood acylcarnitine and urine organic acid analysis were normal. Plasma L-serine was 32 μmol/L at 12 h after fasting, and other amino acids were within normal limits. Brain MRI and EEG were normal. A pure hearing threshold examination revealed no abnormalities. Fundus examination showed mild atrophy in both perioptic discs.

### 3.2 Electrophysiological changes

In patient 1, motor nerve conduction velocity (MNCV) was not elicited in the bilateral median and peroneal nerves. MNCV of ulnar and tibial nerves showed a severe reduction of compound muscle action potentials (CMAP) and a moderate decrease in conduction velocity. Sensory NCV (SNCV) showed that NCV and sensory nerve action potentials (SNAP) were decreased in the upper limbs, and no responses in the bilateral superficial peroneal nerves (Table 1). A needle electromyogram (EMG) showed a chronic and active neurogenic pattern in the muscles of the upper and lower limbs. In patient 2, the latency, velocity, and CMAP of the tested nerves in the upper limbs were within normal limits. MNCV showed that CMAP and velocity of peroneal and tibial nerves decreased asymmetrically. SNCV showed normal variables of NCV and SNAP in the median nerves, and no responses in the superficial peroneal nerves (Table 1). Needle EMG showed a chronic and active neurogenic pattern in the muscles of the lower limb.

**TABLE 1:** Nerve conduction study in the two patients.

Motor nerve	Position	Patient 1			Patient 2		
		MNCV (m/s)	dL (ms)	CAMP (mV)	MNCV (m/s)	dL (ms)	CAMP(mV)
L. median	W-APB	NR (>50.0)	NR (<4.2)	NR (>4.8)	−	−	−
	E-W	NR	NR	NR	−	−	−
R. median	W-APB	NR	NR	NR	−	2.9 (<4.2)	11.6 (>4.8)
	E-W	NR	NR	NR	57.5 (>50.0)	6.9	9.3
L. ulnar	W-ADM	40 (>51.0)	5.3 (<3.1)	0.5 (>5.5)	−	2.7 (<3.1)	6.1 (>5.5)
	E-W	−	−	−	57.9 (>51.0)	6.5	6.1
R. ulnar	W-ADM	39	4.0	0.5	−	−	−
	E-W	−	−	−	−	−	−
L. peroneal	A-EBD	NR (>40.0)	NR (<4.6)	NR (>2.3)	−	4.0 (<4.6)	1.8 (>2.3)
	FH-A	NR	NR	NR	29.9 (>40.0)	14.7	1.1
R. peroneal	A-EBD	NR	NR	NR	−	4.8 (<4.6)	0.9 (>2.3)
	FH-A	NR	NR	NR	35.0 (>40.0)	13.8	0.8
L. tibial	A-AH	34 (>40.0)	5 (<6.1)	0.1 (>3.0)	43 (>40.0)	4.3 (<6.1)	3.1 (>3.0)
R. tibial	A-AH	29	5.7	0.1	45 (>40.0)	3.8 (<6.1)	1.9 (>3.0)
Sensory nerve		SNCV (m/s)	dL (ms)	SNAP (µv)	SNCV (m/s)	dL (ms)	SNAP (µv)
L. median	IIIF-W	40	4.7	4.1	54.3	2.3	13
R. median	IIIF-W	40	4.2	8.3	55.1	2.0	15
L. sup. peroneal	A-L	NR	NR	NR	NR	NR	NR
R. sup. peroneal	A-L	NR	NR	NR	NR	NR	NR

A, ankle; ADM, abductor digiti minimi; AH, abductor hallucis; APB, abductor pollicis brevis; CMAP, compound motor action potential; dL, distal motor latency; E, elbow; EDB, extensor digitorum brevis; FH, fibula head; IIIF, third finger; L, lateral crural region; MNCV, motor nerve conduction velocity; NR, no record; PF, popliteal fossa; SNAP, sensory nerve action potential; SNCV, sensory nerve conduction velocity; and W, wrist. Normal values are given in brackets.

### 3.3 Pathological changes

**FIGURE 3:**
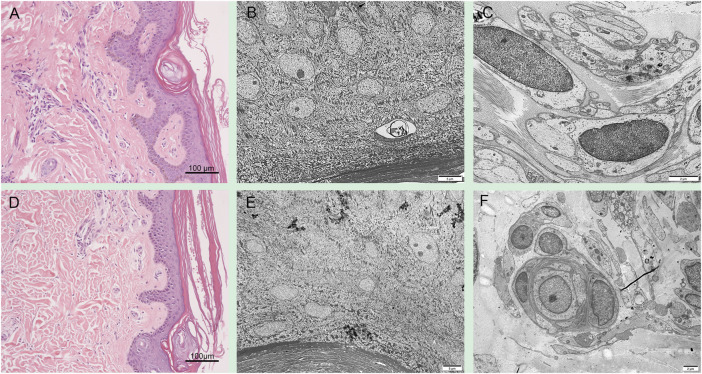
Skin pathology in *PSAT1*-related patients. Skin pathological findings in the patient 1 showed severe orthokeratotic hyperkeratosis and decreased granular layer **(A)**. Electron microscopy revealed a loss of granular layer with vacuolar degeneration **(B)** and unmyelinated axons **(C)**. Histopathological findings in the skin of the patient 2 showed hyperkeratosis in the stratum corneum and loss of granular layer **(D)**. Electron microscopy revealed a loss of granular layer **(E)** and unmyelinated axons **(F)**.

Skin pathological findings in patient 1 showed severe laminar to compact orthokeratotic hyperkeratosis extending in the follicular infundibula and decreased granular layer with follicular plugging (Figure 3A). Electron microscopy revealed a loss of granular layer with vacuolar degeneration (Figure 3B) and unmyelinated axons (Figure 3C). Histopathological findings in the skin of the patient 2 showed hyperkeratosis in the stratum corneum and loss of granular layer with follicular plugging (Figure 3D). Electron microscopy revealed a loss of granular layer (Figure 3E) and unmyelinated axons (Figure 3F).

**FIGURE 4:**
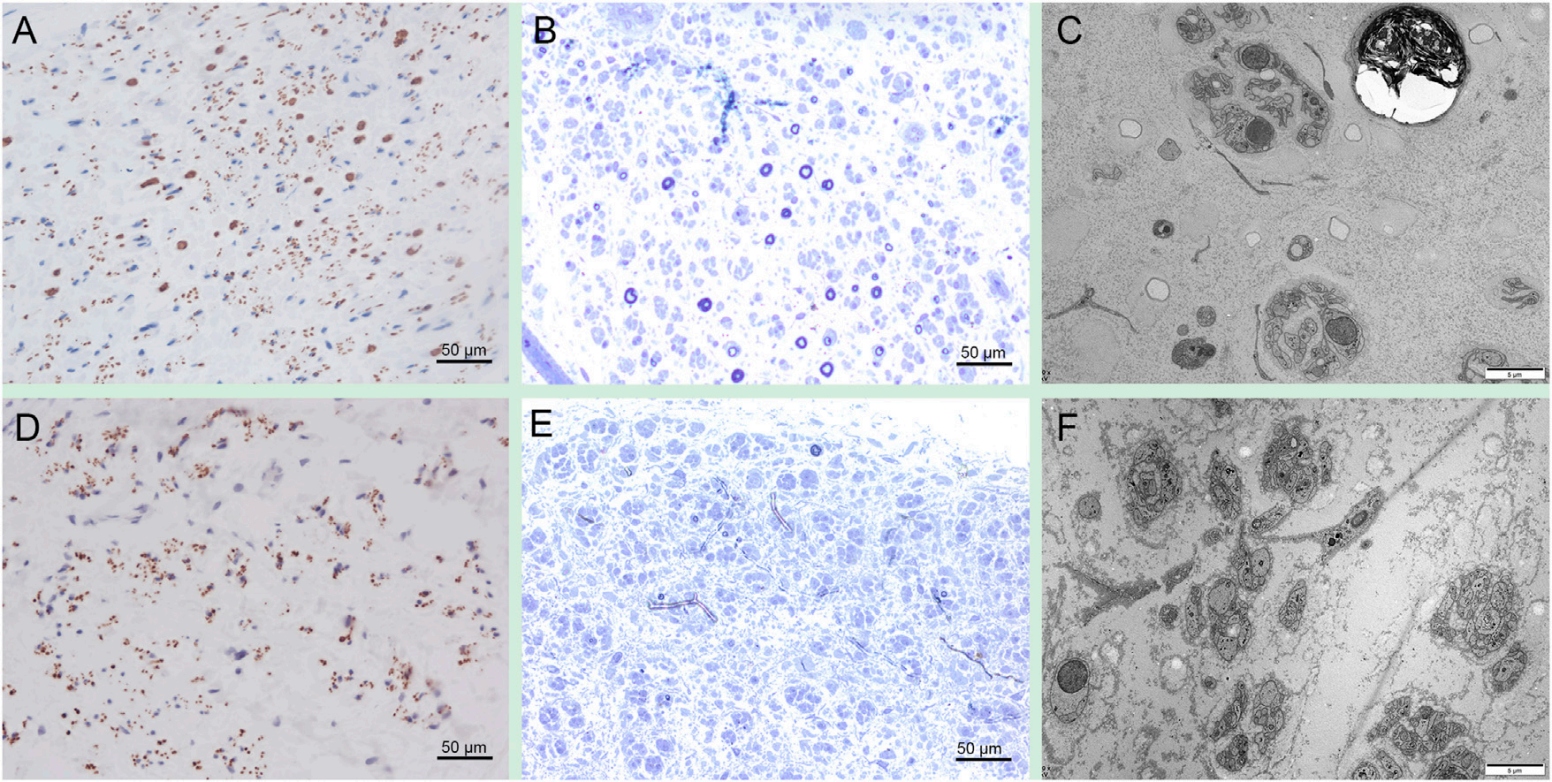
Sural nerve pathology in PSAT1-related patients. Sural biopsy in patient one showed a severe loss of axons on NF immunostaining **(A)**. Toluidine blue staining revealed a severe loss of myelinated axons and a few thin myelinated fibers **(B)**. Electron microscopy occasionally revealed acute axonal degeneration, cavities after axonal degeneration, and severely decreased density of unmyelinated axons with multiple collagen pockets **(C)**. Sural biopsy in patient 2 showed a complete loss of large myelinated fibers and a severe loss of unmyelinated fibers on NF immunostaining **(D)**. Toluidine blue staining revealed a severe loss of axons **(E)**. Electron microscopy revealed a severely decreased density of unmyelinated axons with multiple collagen pockets **(F)**.

Sural nerve biopsy in patient 1 showed a severe loss of axons on NF immunostaining (Figure 4A). Toluidine blue staining revealed a severe loss of myelinated axons and a few thin myelinated fibers, but the absence of active axonal degeneration, regenerative cluster, and onion-like fibers (Figure 4B). Electron microscopy occasionally revealed acute axonal degeneration, cavities after axonal degeneration, and severely decreased density of unmyelinated axons with multiple collagen pockets (Figure 4C). Sural nerve biopsy in patient 2 showed a complete loss of large myelinated fibers and a severe loss of unmyelinated fibers on NF immunostaining (Figure 4D). Toluidine blue staining revealed a severe loss of axons (Figure 4E). Electron microscopy revealed a severely decreased density of unmyelinated axons with multiple collagen pockets (Figure 4F). There were no unusual inclusions seen on electron microscopy.

### 3.4 Genetic mutation

**FIGURE 5:**
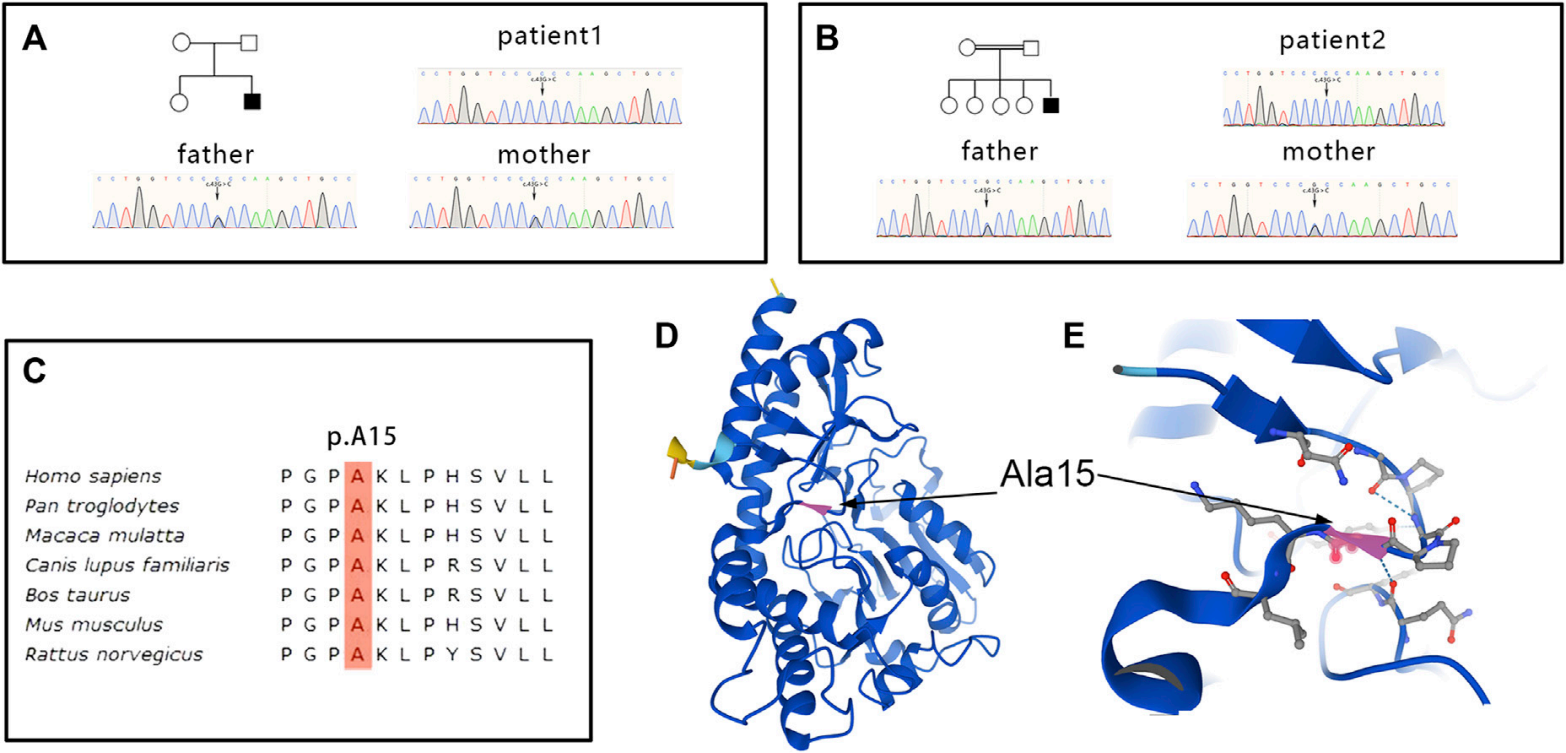
Genetic and structural changes in PSAT. Genetic sequencing revealed a homozygous variant c.43G > C (*p*.A15P)in the *PSAT1* gene in patient 1 **(A)** and 2 **(B)**, and the heterozygous variant came from their parents, respectively. The amino acid at residue 15 is evolutionally highly conserved **(C)**. Residue Ala15 of PSAT is located in a corner of the beta sheet **(D)**. Substitution p.A15Pmight be predicted to disrupt the hydrogen bond between the large side chains of neighboring residues **(E)**.

WES sequencing revealed a homozygous variant c.43G > C (*p*.A15P) in the *PSAT1* gene in both patients 1 and 2 (Figures 5A, B). The heterozygous variant came from their parents, respectively, indicating a family cosegregation. The variant had been previously reported in another patient (Debs, et al., 2021), and had a low allele frequency (12/367,166) in the gnomAD database (http://gnomad.broadinstitute.org). A mutation (c.44C > T; *p*.A15V) at the same site leading to substitution for other amino acids has been confirmed to be pathogenic (Debs, et al., 2021). A homology search in different species demonstrated that the amino acid at residue 15 is evolutionally highly conserved (Figure 5C). The variant was predicted to be damaged by several *in silico* tools. Of note, functional studies in yeast revealed that the growth rate of yeast with *p*.A15P mutant was significantly below that of the wild type, indicating a loss-of-function of PSAT with *p*.A15P mutant. (Sirr, et al., 2020). Collectively, the significance of the variant was evaluated as pathogenic according to the ACMG criteria (PS1+PS3+PM5+PP1) (Richards et al., 2015). No other variants associated with ichthyosis or neuropathy were found in the genetic screening.

### 3.5 Treatment follow-up

Patient 1 was treated with oral L-serine (300 mg/kg/day) and glycine (300 mg/kg/d). Three months later, the skin symptoms showed significant improvement with almost normal skin color (Figure 2C). The patient also had increased muscle strength manifesting an improved ability of climbing stairs and long-distance walking. However, sensory impairment, especially deep sensory dysfunction, had not been alleviated. Patient 2 was treated with oral L-serine (300 mg/kg/day) and glycine (300 mg/kg/d). Five months later, the skin symptoms were completely resolved (Figure 2D). Muscle strength grade was 5/5 in the upper limbs, 5/5 in the proximal lower limbs, 4/5 in the foot dorsiflexion, and 4+/5 in the foot plantar flexion. Pain and temperature sensations were reduced at the soles and toes. Deep tendon reflexes were reduced at the knee and ankles. Horizontal and vertical nystagmus was relieved. Unfortunately, the level of plasma L-serine and glycine was not evaluated in the two patients again.

### 3.6 Literature review

In addition to the two patients in this study, *PSAT1*-related serine deficiency disorder have been reported in other 29 individuals including 13 males, 15 females, and one unidentified gender died *in utero* (Table 2) (Hart, et al., 2007; Acuna-Hidalgo, et al., 2014; Brassier et al., 2016; El-Hattab, et al., 2016; Glinton et al., 2018; Ni et al., 2019; Abdelfattah et al., 2020; Debs, et al., 2021). Fourteen patients came from consanguineous families. Eighteen individuals died at birth or within 1 month of birth, and the rest were born at full term. The age of onset ranged from intrauterine to 4 years. The presenting symptoms in the majority of patients included epilepsy and skin changes. The main clinical symptoms showed microcephaly in 24 patients (24/26, 92.3%), intrauterine growth restriction in 11 patients (11/15, 73.3%), ichthyosis in 21 patients (21/24, 87.5%), and epilepsy in 2 patients (2/10, 20.0%). The *PSAT1* variants included c.43G > C (5/62 alleles, 8.1%), c.44C > T (1/62 alleles, 1.6%), c.delG107 (2/62 alleles, 3.2%), c.129T > G (6/62 alleles, 9.7%), c.296C > T (10/62 alleles, 16.1%), c.299A > C (2/62 alleles, 3.2%), c.432delA (1/62 alleles, 1.6%), c.497C > T (1/62 alleles, 1.6%), c.1A > G (4/62 alleles, 6.5%), c.536C > T (2/62 alleles, 3.2%), and c.del1023_1027insAGACCT (2/62 alleles, 3.2%).

**TABLE 2:** Clinical features and treatment outcomes in all reported patients with *PSAT1*-related serine deficiency disorder.

Variables	This study		Debs et al. (2021)	Hart et al. (2007)		Brassier et al. (2016)	Glinton et al. (2018)	Acuna-Hidalgo et al. (2014)			
	1	2	3	4 (Patient 1)^$^	5 (Patient 2)	6 (Patient 2)	7 (Patient 4)	8 (Patient 1)	9–11 (Patient 2,4,5)	12 (Patient 3)	13 (Patient 6)
Mutation	c.43G > C (*p*.A15P)	c.43G > C (*p*.A15P)	c.497C > T (*p*.T156M)	c.299A > C (*p*.D100A)	c.299A > C (*p*.D100A)	c.129T > G (*p*.S43R)	c.432delA (*p*.D145Mfs*49) c.44C > T (*p*.A15V)	c.del1023_1027del/insAGACCT (*p*.R342Dfs*6)	c.296C > T (*p*.A99V)	c.536C > T (*p*.S179L)	c.296C > T (*p*.A99V) c.536C > T (*p*.S179L)
			c.43G > C (*p*.A15P)	c.delG107 (*p*.G36Afs*5)	c.delG107 (*p*.G36Afs*5)						
Gender	Male	Male	Female	Male	Female	Male	Female	Male	Two males and one female	Male	Female
Age of onset	Infancy	4 years	Infancy	2 weeks	2 weeks	1 month	2 weeks	Died	Died	Died	Died
Consanguineous	No	Yes	No	No	No	Yes	No	Yes	Yes	Yes	No
IUTR	No	No	Yes	Yes	No	Yes	Yes	Yes	Yes	Yes	Yes
Gestation	Full term	Full term	Full term	Full term	Full term	NR	Full term	Died	Died	Died	Died
Birth weight	Normal	Normal	8th percentile	9th percentile	9th percentile	NR	Normal	NR	NR	NR	NR
Birth length	Normal	Normal	NR	NR	NR	NR	NR	NR	NR	NR	NR
HC	Normal	Normal	Microcephaly	Microcephaly	Microcephaly	Microcephaly	Microcephaly	Microcephaly	Microcephaly	Microcephaly	Microcephaly
Epilepsy	No	No	No	Yes	No	No	No	NR	NR	NR	NR
Ichthyosis	Yes	Yes	Yes	NR	NR	NR	NR	Yes	Yes	Yes	Yes
Blood serine μmol/L (normal)	66 (65–180)	32 (65–180)	21 (63–187)	51 (60–300)	30 (50–350)	34 (111–165)	21 (83–212)	NR	NR	NR	NR
CSF serine μmol/L (normal)	NR	NR	3.8 (18.3–52.2)	18 (35–80)	5 (35–80)	7 (27–70)	10 (22–61)	NR	NR	NR	NR
Brain MRI	Normal	Normal	Normal	Abnormal	Normal	Abnormal	Abnormal	NR	NR	NR	NR
EEG	Normal	Normal	Normal	Abnormal	Normal	Abnormal	Abnormal	NR	NR	NR	NR
Age of Treatment	19	17	38	11 weeks	24 h	3 months	5 months	NR	NR	NR	NR
Treatment (mg/kg/d)	300/Ser, 400/Gly (3 months)	300/Ser, 400/Gly (5 months)	100/Ser (2 years)	500/Ser, 200/Gly (5 months)	500/Ser, 200Gly (3 years)	400–950/Ser (5.5 years)	500/Ser, 200/Gly	NR	NR	NR	NR
Treatment outcomes	Normal skin, elevated strength	Normal skin, elevated strength	Normal skin and hypertension	Died	Normal development	Improved spasticity, no progress	Weight gain and irritability improved	NR	NR	NR	NR

Variables	El-Hattab et al. (2016)	Ni et al. (2019)	Abdelfattah al. (2020)								
	14(Patient 2)	15–16(Family 1)	17–18	19–20	21–22	23	24–26	27	28	29	30–31
Mutation	c.296C > T (*p*.A99V)	c.208T *>* A (Y70N)	c.1A > G	c.129T > G (*p*.S43A)	c.181C > T (*p*.A61T)	c.235G > T (*p*.G79T)	c.296C > T (*p*.A99V)	c.733T > C (*p*.C245A)	c.463G > C,c.8701G > T (*p*.Glu155Gln)	c.8701G > T	c.955delAp. (Arg319Aspfs*14)
		c.1024C *>* T (R342W)			c.296C > T (*p*.A99V)						
Gender	Male	Male	Female	Male	Male/female	Female	Two males/one female	Male	Female	Female	Male
Age of onset	Infancy	Infancy	Died	Infancy	Died/infancy	Died	Died	Died	Died	Infancy	Died
Consanguineous	Yes	No	Yes	Yes	No	Yes	No	Yes	No	No	No
IUTR	Yes	No	NR	NR	NR	NR	NR	NR	NR	NR	NR
Gestation	Full term	Full term	Full term	Full term	Died	Died	Full term	Died	Full term	Stillborn	Died
Birth weight	NR	<3th percentile	<3th percentile	<3th percentile	<3th percentile	<3th percentile	<3th percentile	<3th percentile	<3th percentile	NR	<3th percentile
Birth length	NR	NR	<3th percentile	<3th percentile	<3th percentile	NR	<3th percentile	<3th percentile	NR	NR	<3th percentile
HC	Microcephaly	Microcephaly	Microcephaly	Microcephaly/NR	NR/microcephaly	NR	Microcephaly	Microcephaly	Microcephaly	NR	NR/microcephaly
Epilepsy	Yes	No	NR	NR	NR	NR	NR	NR	NR	NR	NR
Ichthyosis	Yes	Yes	Yes	Yes/NR	NR	No	Yes	Yes	No	No	Yes
Blood serine μmol/L (normal)	NR	NR	NR	NR	NR	NR	NR	NR	NR	NR	NR
CSF serine μmol/L (normal)	NR	NR	NR	NR	NR	NR	NR	NR	NR	NR	NR
Brain MRI	NR	NR	NR	NR	NR	NR	NR	NR	NR	NR	NR
EEG	NR	NR	NR	NR	NR	NR	NR	NR	NR	NR	NR
Age of Treatment	NR	NR	NR	NR	NR	NR	NR	NR	NR	NR	NR
Treatment (mg/kg/d)	NR	NR	NR	NR	NR	NR	NR	NR	NR	NR	NR
Treatment outcomes	NR	NR	NR	NR	NR	NR	NR	NR	NR	NR	NR

IUGR, intrauterine growth restriction; HC, head circumference; NR, indicates was not described in these reports; Yes/No, indicates the presence of symptoms; $, case number described in report; MRI, magnetic resonance imaging; CSF, cerebrospinal fluid; EEG, electroencephalogram.

Most patients with serine deficiency disorders presented with severe developmental delay and neurologic symptoms at birth, even *in utero*, while a few patients could show a milder phenotype mainly characterized by ichthyosis and peripheral neuropathy. Up to now, this phenotype were identified in only five patients, including three caused by *PSAT1* (one reported and two in this study), one by *PHGDH*, and one by *PSPH* (Méneret et al., 2012; Byers et al., 2016; Debs, et al., 2021). The patients (three males and two females) initially presented with ichthyosis, cataract, or seizure. Two of five patients had intrauterine growth restriction; three of five had mental retardation; four of five had multidirectional nystagmus; five of five had peripheral neuropathy. All patients could grow up to be able to live, and show great improvement after treatment (Table 3). In detail, a 31-year-old male patient presented with congenital cataract, and length-dependent symmetrical sensorimotor neuropathy since 8 years old. His serine deficiency was associated with compound mutations of c.1471C > T (*p*.R491Y) and c.1273G > A (*p*.V425M) in the *PHGDH* gene (Méneret, et al., 2012). A 38-year-old female patient showed mild absence of seizures and intellectual disability at age 4, and progressive myeloneuropathy since 19 years old. Her serine deficiency was caused by compound mutations of c.131T > G (*p*.V44G) and c.421G > A (*p*.G141S) in the *PSPH* gene (Byers, et al., 2016). A 38-year-old female patient showed intrauterine growth restriction and ichthyosis at birth, peripheral neuropathy, and contractures since 13 years old. Her serine deficiency was caused by compound mutations of c.467C > T (*p*.T156M) and c.43G > C (*p*.A15P) in the *PSAT1* gene (Debs, et al., 2021).

**TABLE 3:** Clinical features and treatment outcomes in patients with prominent peripheral neuropathy.

Variables	This study		Debs et al. (2021)	Méneret et al. (2012)	Byers et al. (2016)
	1	2	3	4	5
Gene	PSAT1	PSAT1	PSAT1	PHGDH	PSPH
Mutation	c.43G > C (*p*.A15P)	c.43G > C (*p*.A15P)	c.497C > T (*p*.T156M)	c.1471C > T (*p*.A491T)	c.131T > G (*p*.V44G)
			c.43G > C (*p*.A15P)	c.1273C > A (*p*.V425M)	c.421G > A (*p*.G141S)
Gender	Male	Male	Female	Male	Female
Intrauterine growth restriction	No	No	Yes	No	Yes
Consanguineous	No	Yes	No	No	No
Symptoms at birth	Ichthyosis	No	Microcephaly and ichthyosis	Congenital cataract	Absence seizure
Mental retardation	No	No	Yes	Yes	Yes
Epilepsy	No	No	No	No	Yes
Neuropathy	Motor and sensory (16 years old)^a^	Motor and sensory (17 years old)	Motor (13 years old)	Motor and sensory (8 years old)	Motor and sensory (19 years old)
Ichthyosis	Yes (birth)	Yes (4 years old)	Yes (birth)	No	No
Contracture	Yes (18 years old)	No	Yes (20 years old)	Yes (30 years old)	Yes (32 years old)
Ocular sign	Yes	Yes	Yes(infancy)	Yes	No
Hearing symptoms	Yes	No	Yes(20 years old)	No	No
Electrophysiological examination	Abnormal (motor and sensory)	Abnormal (motor and sensory)	NR	Abnormal (motor and sensory)	Abnormal (motor and sensory)
Skin examination	Abnormal	Abnormal	Abnormal	Normal	Abnormal
Skin biopsy	Abnormal	Abnormal	NR	NR	NR
Muscle biopsy	NR	NR	Abnormal	NR	NR
Nerve biopsy	Abnormal	Abnormal	Axonal loss	NR	NR
Blood serine (normal)	66 μmol/L (65–180)	32 μmol/L (65–180)	21 μmol/L (63–187)	0.35 mg/dL (0.66–2.26)	31 mcmol/L (60–170)
CSF serine (normal)	NR	NR	3.8 μmol/L (18.3–52.2)	0.14 mg/dL (0.21–0.43)	NR
Brain MRI	Normal	Normal	Normal	Normal	Normal
EEG	Normal	Normal	Basically normal	Normal	Epileptic discharge
Age of Treatment Onset (years old)	19	17	38	31	38
Treatment (mg/kg/d)	300/Ser, 400/Gly (3 months)	300/Ser, 400/Gly (5 months)	100/Ser(2 years)	80/Ser(3 months)	80/serine (4 months)
Treatment outcomes	Normal skin and elevated strength	Normal skin and elevated strength	Normal skin and hypertension	Improved walking ability	Reduced paresthesia

a Age of onset described in the text in parentheses; NR, unavailable; Yes/No: indicates the presence of symptoms; MRI, magnetic resonance imaging; CSF, cerebrospinal fluid; EEG, electroencephalogram.

## 4 Discussion

In this study, the two patients initially showed infancy- or childhood-onset ichthyosiform lesions that were mainly characterized by flaky and red skin on the trunk and limbs, which would be aggravated or alleviated with the changes in temperature and seasons. The juvenile-onset neuropathy was characterized by length-dependent axonal sensorimotor neuropathy. Sural nerve biopsy indicated a severe axonal neuropathy, which was consistent with the electrophysiological changes. Molecular investigation revealed a pathogenic homozygous mutation in the *PSAT1* gene. Oral treatment with serine and glycine improved the symptoms of ichthyosis and neuropathy. Collectively, the clinical characteristics were very similar between the two patients and were partially in accordance with the phenotype related to a milder form of serine deficiency disorders.

Similar to the other serine deficiency disorders, the *PSAT1*-related phenotype showed great clinical heterogeneity from severe type of Neu-Laxova syndrome to childhood-onset neurodegenerative disease (El-Hattab, et al., 2016). Recently, an adult female patient sequentially presented with intrauterine growth restriction, microcephaly and ichthyosis at birth, intellectual disability and esotropia in childhood, clumsy gait, and limb contractures in adolescence, and peripheral neuropathy and primary ovarian insufficiency in adulthood (Debs et al., 2021). The case report extended the *PSAT1*-related clinical spectrum into a very late-onset milder multiple system degenerative phenotypes. Of note, a few patients with *PHGDH* or *PSPH* mutations prominently showed juvenile-onset peripheral neuropathy and mild neurodevelopment disorders. Our patients also had juvenile-onset neuropathy that was similar to the phenotype of the previously described patients, while no symptoms of developmental delay including intrauterine growth restriction, microcephaly, intellectual disability, seizure, and esotropia were observed in our patients. In this study, the patients with a phenotypic combination of infancy-onset ichthyosis and juvenile-onset neuropathy may represent an overall milder and different form of serine synthesis defect secondary to PSAT deficiency. Juvenile-onset peripheral neuropathy might be the mildest end of serine deficiency disorders.

The underlying pathogenesis of peripheral neuropathy in the patients is not well elucidated. Previous studies have indicated that limited serine availability may result in a great decrease in physiological sphingolipids (El-Hattab, 2016) or phospholipids (Glinton, et al., 2018), which are biosynthesized using serine as a substrate. Another possible explanation is the increase of atypical sphingolipids, such as 1-deoxysphingolipids, which are synthesized by a noncanonical substrate alanine or glycine, instead of serine, catalyzed by serine palmitoyl transferase (SPT), and plays a neurotoxic role in the pathogenesis of neuropathy (Penno et al., 2010; Ferreira et al., 2018). Of note, SPT is an enzymatic complex composed of SPTLC1 and SPTLC2 subunits (Yard et al., 2007). Mutations in *STPLC1* and *SPTLC2* have been associated with hereditary sensory and autonomic neuropathy types IA and IC (HSAN1A and HSAN1C), respectively (Rotthier et al., 2010; Murphy et al., 2013; Oswald et al., 2015). Sural nerve pathology in our patients showed severe loss of myelinated and unmyelinated fibers, which was similar to the neuropathological changes of HSAN1A and HSAN1C and indicated an axonal degeneration caused by the accumulation of neurotoxic metabolisms. The neuropathy associated with *SPTLC1* mutation exhibited some improvement after supplementation with oral L-serine (Fridman et al., 2019), which also partially improved the symptoms of sensorimotor and autonomic neuropathy in our *PSAT1*-related patients. Collectively, the neuropathic phenotype in serine-deficient patients may be explained by the combination of nerve tissue dysfunctions due to a shortage of sphingolipids and the neurotoxic effect of atypical sphingolipids. Additionally, some patients with *PSAT1* mutations showed sensorineural hearing loss and nystagmus that was usually attributed to dysfunctions of auditory and vestibular nerves or their ascending pathways. In this study, we found that impairments of semicircular canals might be partially involved in the pathogenesis of nystagmus.

An ichthyosis is a heterogeneous group of inherited cornification disorders characterized by generalized dry skin, scaling, and/or hyperkeratosis (Miao et al., 2021). The ichthyosiform lesion has been described in more than 50% of individuals with serine deficiency disorder, while the pathogenesis of ichthyosis is not well clear (El-Hattab, et al., 2016). Serine is a vital component for the enzyme active site of serine proteases, which are a group of enzymes that play a major role in cleaving profilaggrin to filaggrin. Filaggrin peptides are key proteins in facilitating epidermal differentiation and can aggregate the keratin filaments forming the stratum corneum (Holbrook et al., 1982; El-Hattab, et al., 2016). Skin pathological changes in our patients showed the hyperkeratosis of the stratum corneum and the loss of granular layer, which seemed to be consistent with the pathogenic mechanism. However, in adult individuals with serine deficiency disorder, only *PSAT1*-related patients presented with ichthyosis, but not in *PHGDH*- and *PSPH*-related patients. This raises questions about the above serine deficiency hypothesis. Therefore, other explanations should be supplemented in pathogenic mechanisms. Since the extracellular lipid of stratum corneum contains lots of sphingolipids, the stratum corneum will be disturbed by sphingolipid shortage due to serine deficiency (Holleran et al., 2006). Deficient synthesis of activated tetrahydrofolate due to serine deficiency can affect epidermal cell division and that in turn may contribute to skin pathology (El-Hattab, et al., 2016). In addition, our skin pathology revealed a severe loss of unmyelinated axons, which may be involved in the skin’s nutritional and dysfunctional disorders.

The PSAT1 enzyme is a pyridoxal phosphate (PLP) dependent homodimer that is highly conserved from bacteria to humans (Baek et al., 2003). All mutations of *PSAT1* associated with serine deficiency disorder have been identified as loss of function, which leads to the defect in the serine biosynthesis pathway (Acuna-Hidalgo, et al., 2014). The variant at residue Ala15 of PSAT was reported in other two patients with serine deficiency disorders (Glinton, et al., 2018; Debs, et al., 2021). The Ala15 is located in a corner of the beta sheet (Figure 5D), and substitution *p*.A15P might be predicted to disrupt the hydrogen bond between the large side chains of neighboring residues (Figure 5E). Functional experiments in yeast have demonstrated that the *p*.A15P mutant exhibited a reduced growth compared to wild-type *PSAT1* in the absence of serine (Sirr, et al., 2020). Collectively, the *p*.A15P would be a disease-causative mutation, which played a pathogenic role through loss of function. The variability in the phenotypes of serine biosynthesis defects had been suggested to be related to the degree of the residual enzyme activity but not to the level of plasma serine concentration (de Koning and Klomp, 2004; Brassier, et al., 2016). Our patients with the *p*.A15P presented with a somewhat milder phenotype, and the first patient had a plasma serine concentration marginally above the lower limit of the reference range. A mild decrease in plasma serine concentration was previously observed in adult-onset *PHGDH*-related patients; conversely, a similar level of plasma serine was also reported in severe infantile phenotype (Tabatabaie et al., 2009). Therefore, plasma levels of serine could be at the lower end of the normal range, especially in nonfasting samples, in a few patients with serine deficiency disorders (Hart, et al., 2007; Tabatabaie et al., 2011; Debs, et al., 2021). The mild phenotype may not be only predicted from the biochemical or molecular analysis, and dynamic measurements of serine levels in plasma and cerebrospinal fluid (CSF) are needed to better explain the development of clinical symptoms in our patients. Accurate assessment of serine levels usually requires measurement of metabolite concentrations in CSF and comparison with age-matched controls (Moat et al., 2010). Of note, patient 2 had a slight increase in CSF protein level that might be associated with the chronic degeneration of spinal nerve roots or spinal cord, while chronic inflammatory polyradiculoneuropathy could be distinguished according to the electrophysiological and pathological findings.

*PSAT1*-related serine deficiency is potentially treatable by oral L-serine alone or combined with glycine with varying degrees of success (Debs, et al., 2021). At present, there is no consistent recommendation on the therapeutic dose of serine, but it has been unquestionably agreed to start adding serine as soon as possible. Previous observations revealed that patient diagnosed at birth and treated with L-serine and glycine in the first 24 h of life was neurologically normal at the age of 3 years (Hart et al., 2007). Our patients showed a completed recovery on the skin lesions but presented with very mild improvement of neuropathic symptoms after administering L-serine and glycine. This finding indicates that the ability of tissue regeneration determines the efficacy of serine drug therapy. Early diagnosis and treatment are very important for serine deficiency disorders, especially before irreversible neurologic damage occurs.

In summary, serine deficiency disorders include a broad clinical spectrum, and the skin and nervous system may be the main and consistent target organs. Our patients presented with infancy- or childhood-onset ichthyosis and juvenile-onset peripheral neuropathy, which expands the clinical phenotype of *PSAT1*-related serine deficiency disorder. The pathological changes and regenerative ability of skin and peripheral nerves determine their response to serine therapy. Therefore, early diagnosis and treatment are vital to the prognosis of *PSAT1*-related neuropathy as a treatable disease.

## Data Availability

The data has been deposited in the GenBank database (BankIt2600251) https://www.ncbi.nlm.nih.gov/nuccore/ON936865, the accession number(s) are 936865-936870.
